# Changes in the activity of adult stages of *Dermacentor reticulatus* (Ixodida: Amblyommidae) induced by weather factors in eastern Poland

**DOI:** 10.1186/1756-3305-7-245

**Published:** 2014-05-28

**Authors:** Alicja Buczek, Katarzyna Bartosik, Zbigniew Zając

**Affiliations:** 1 Department of Biology and Parasitology, Medical University, Radziwiłłowska 11 St., 20-080 Lublin, Poland

**Keywords:** *Dermacentor reticulatus*, Diapause, Winter activity, Eastern Poland

## Abstract

**Background:**

The host-seeking activity in *Dermacentor reticulatus* ticks undergoes rhythmical changes correlated with environmental conditions. Therefore, this study is focused on investigating the activity of adult stages of the species during weather changes occurring in winter months in eastern Poland, i.e. a period of tick diapause.

**Methods:**

*D. reticulatus* ticks were collected in a meadow ecosystem near Lublin (eastern Poland, 51°36'N, 22°58'E) between the third decade of November 2011 and the third decade of January 2012. During each collection, temperature and humidity were measured at the soil surface and at a height of 25 cm and the differences (delta) in the parameters between the two measurement points were calculated.

**Results:**

During one hour of our observation, from 0 to 42 specimens were collected, with the greatest numbers (25-40 specimens) between late November and mid- December. The activity of adult *D. reticulatus* (females and males in total) depended on soil and air temperature (r = -0.6986, p < 0.05). Soil and air humidity did not exert an impact on the questing behaviour of adult stages. In turn, the greater the moisture delta between these two measurement points was, the greater the activity of the adult tick stages was observed.

Our investigations have demonstrated differences in the questing behaviour between *D. reticulatus* females and males correlated with environmental conditions. The temperatures of soil and air increased the migratory activity in females but did not affect migration of male ticks. In turn, the deltas of temperatures (r = -0.6986, p < 0.05) and humidity (r = 0.6829, p < 0.05) did not have a statistically significant effect on stimulation of female activity but they induced significant changes on the behaviour of males, for which we found a highly negative correlation between the number of active specimens and the temperature delta (r = -0.7276, p < 0.05) and a highly positive correlation with the humidity delta (r = 0.8199, p < 0.01).

**Conclusions:**

Weather changes can be accompanied by activity of adult *D. reticulatus* even in their winter diapause period. Therefore, there is an increased threat to human and animal health posed by tick-borne diseases.

## Background

The meadow tick *Dermacentor reticulatus* (Fabricius, 1794) has a large geographical range across the temperate zone, where it usually occurs in open and mid-forest meadow ecosystems. It mainly infests wild and domestic animals [1-5], although specimens of this tick had been found attached to human skin [6-8]. *D. reticulatus* is a vector of many pathogens, e.g. *Babesia canis*[9], *Babesia microti*[10], *Anaplasma phagocytophilum*[11], *Francisella tularensis*[12], spotted fever group rickettsiae [13,14] and the tick-borne encephalitis virus [15]. In some areas, the species may colonize the same habitats and infest the same animal species as *Ixodes ricinus* (L.) [16]; unpubl. data; this may contribute to interspecific transmission of tick-borne pathogens and maintenance of natural foci of human and animal infectious diseases.

The seasonal activity of adult *D. reticulatus* has been repeatedly studied in eastern [17-19], central [20,21] and western [22] Europe. Although the abundance and dynamics of the activity of these ticks vary, two activity peaks have always been reported: in spring and autumn. As in the case of other exophilic tick species, the seasonal activity of *D. reticulatus* depends on environmental factors, particularly the photoperiod and temperature, and on the occurrence of tick hosts and their biological rhythms [20,23].

According to the previous observations in the area of Poland, the activity of this tick species begins in mid-March at the earliest [17,19,23] and ends in early December at the latest [19]. This paper presents untypical winter locomotor activities in adult *D. reticulatus* in eastern Poland.

## Methods

### Study area

The investigations were carried out in an area of 4,000 m^2^, 17 km away from Lublin (eastern Poland, 51°36'N, 22°58'E). The locality of *D. reticulatus* was situated on a meadow close to a large mixed forest complex dominated by pines and alders.

The study area is located in the temperate zone with a climate that is transitional between oceanic and continental. On average, the first snow cover appears in this area in the third decade of November and persists for a short period. The permanent snow cover, persisting for over 7 days appears in the second half of December. On average, it persists for 60 days. The mean temperatures of November, December, January, and February are 2.6, -1.3, -3.5 and 2.6°C, respectively [24].

### Tick collection

Ticks were collected between the third decade of November 2011 and the third decade of January 2012. The choice of study days depended on weather conditions. No collection of ticks was arranged after snowfall, rainfall, heavy rains, or on wet snowmelt days.

In the investigations, the flagging method was used, which involved sweeping vegetation with a 1 m^2^ white flannel cloth attached to a bamboo stick. After every 2-3 paces, the cloth was viewed and adult *D. reticulatus* ticks attached to it were counted (females and males separately) and released in their locality. Each collection round was carried out by one person in a 20 × 20 m experimental plot and lasted for an hour. Ticks were collected during the diurnal activity peaks for this species in eastern Poland, i.e. between 1 p.m. and 2 p.m. [25].

Concurrently, during each collection round, temperature with an accuracy of 1°C and relative humidity with an accuracy of 1% were measured on the soil surface and at the height of 25 cm. Based on the measurements, the temperature and humidity deltas were calculated, which is the difference between their values at the soil surface and in the air. For all the study days, the length of the photoperiod was calculated for the geographical position of the tick locality, based on the information about the sunrises and sunsets according to CET (Central European Time) (http://www.calendar.k-ce.pl).

### Statistical analysis

The analysis of the results was performed using the STATISTICA package for Windows 9.0. The differences in the distribution of the results were analysed with the Spearman rank correlation test. Test probability at the level p < 0.05 was assumed significant, and p < 0.01 was regarded as highly significant.

## Results

The present study demonstrated that adult *D. reticulatus* were active between November 11, 2011 and January 7, 2012. A total of 291 specimens, including 168 females and 123 males, were collected (Table 1). The high activity of ticks persisted from the third decade of November and mid-December. During that period, 20-42 specimens/1 hr were collected. Females dominated in the collection from early to mid-December, which was followed by a decrease in their activity, compared to the activity of males. Higher activity of males was reported between mid- and late December. At the beginning of January, only single specimens were collected, among which females were predominant.

**Table 1:** **Total gender number of *Dermacentor reticulatus *** **ticks collected during particular collections in 2011 and 2012 in context of temperature and relative humidity**

**Date**	**F**	**F (%)**	**M**	**M (%)**	**Total tick no.**	**Temp °C D**	**Temp °C G**	**Temp.**	**RH % D**	**RH % G**	**RH**	**Day length**
23.11.11	18	72	7	28	25	9.2	9.8	0.6	55.5	52	2.5	8h33’
25.11.11*	13	65	7	35	20	7.4	6.9	0.5	80	77	3	8h28’
	19	59.4	13	40.6	32	7.4	6.9	0.5	80	77	3	8h28’
30.11.11*	13	50	13	50	26	5.4	6.1	0.7	83.5	70.5	13	8h16’
	20	58.8	14	41.2	34	5.4	6.1	0.7	83.5	70.5	13	8h16’
02.12.11*	29	69	13	31	42	8.1	8.2	0.1	76.1	63.5	12.6	8h12’
	21	55.3	17	44.7	38	8.1	8.2	0.1	76.1	63.5	12.6	8h12’
16.12.11*	10	38.5	16	61.5	26	5.2	5.1	0.1	82.5	71	11.9	7h54’
	16	50	16	50	32	5.2	5.1	0.1	82.5	71	11.9	7h54’
23.12.11*	5	50	5	50	10	4.9	3.9	1	80.9	75.9	5	7h52’
07.01.12	4	66.7	2	33.3	6	2.5	-	-.	-	-	-	8h06’
26.01.12	0	0	0	0	0	3.6	4.4	0.8	63	62	1	8h26’

F- females, M-males- no data, Temp. G- temperature measured on the soil surface, Temp. D- temperature measured at the height of 25 cm above the ground, RH- relative humidity; *- ticks were collected simultaneously by two persons at the same time (between 1 p.m. and 2 p.m.) in the separate, adjacent 20 × 20 m experimental plots.

Triangle- the difference between parameter values at the soil surface and in the air (delta).

During the study, the mean soil and air temperatures were 6.03 ± 2.00°C and 6.43 ± 1.8°C, respectively (Table 1). The Spearman’s test confirmed the highly significant positive correlations between the number of females and the soil surface temperature (r = 0.8254, p < 0.01) and air temperature (r = 0.7724, p < 0.01). In the case of the total number of the specimens (both females and males), there was a relatively high correlation with the soil-level temperature (r = 0.6254, p < 0.01), but no correlation with the air temperature (r = -0.5115, p > 0.05) was found. No significant correlations were found between the temperature of the soil (r = 0.3968, p > 0.05) and air (r = 0.2116, p > 0.05) and the activity of males. For the temperature delta, a highly significant negative correlation with the number of males (r = -0.7418, p < 0.01), and a significant correlation with the activity of both genders in total (r = -0.6575, p < 0.05) was reported. In turn, the correlation between the temperature delta and the activity of females was not significant (r = -0.5294, p > 0.05).

Humidity measured at the ground level (76.69 ± 9.15%) and air humidity (68.54 ± 7.64%) did not have a significant effect on the number of females (r = -0.1149, p > 0.05; r = -0.2069, p > 0.05), males (r = 0.4070, p > 0.05; r = 0.0581, p > 0.05), and the total number of adult *D. reticulatus* (r = 0.1659, p > 0.05; r = -0.1014, p > 0.05). The humidity delta was significantly positively correlated with the number of males (r = 0.8199, p < 0.01) and the number of both females and males (r = 0.6912, p < 0.05). In turn, in these investigations the activity of females (r = 0.2222, p > 0.05), males (r = -0.2509, p > 0.05), and the total number of adult ticks (r = -0.0565, p > 0.05) did not depend on the length of the day.

## Discussion

Diapause is a period of arrested development and activity in ticks in conditions in which the effect of environmental factors exceeds ticks’ adaptive abilities. Among the abiotic factors, the greatest effect on the seasonality of tick behaviour is exerted by the photoperiod length and temperature. Diapause is neurohormonally and hormonally stimulated and is significant in seasonal regulation of the tick developmental cycle. As indicated in our 11-year-long field research, diapause in adult *D. reticulatus* in eastern Poland begins in late November or early December [19], unpubl. data. The present investigations have shown that the high activity of these ticks can persist even throughout December, during the shortest days of the year at soil and air temperatures ca. 5°C (Table 1). It is comparable to the autumn activity peak of adult forms of the species reported in the previous studies from eastern Poland; this was 62.9 ± 54.3 specimens/2 hr of collection and exceeded the spring peak activity (32.5 ± 24.7 specimens/2 hr of collection) [19]. We found active adult ticks in January, at air temperatures of 2.5°C; however, they were not found at higher temperatures (soil/air temperature 3.6 and 4.4°C, respectively), which may be explained by persistence of the snow cover in the habitat, preventing tick emergence, although, unlike in the case of an exposed surface, the snow cover and its properties (albedo, thermal conductivity) have a beneficial impact on the radiation and heat balance of the active surface [26].

This study has shown differences in the questing behaviour of *D. reticulatus* males and females caused by temperature during the winter season. The activity of females increased in parallel with rising soil and air temperature, although the difference between temperatures measured at the respective points did not exhibit a stimulating effect on these specimens. In turn, in the case of males, the bigger the temperature delta was the lowest activity they exhibited. According to Alekseev and Dubinina [26], the soil temperature gradient 1-1.5 cm below leaf litter and soil surface temperature seem to be the main physical parameters triggering migration of *Ixodes persulcatus* Schulze from the leaf litter. As many as 60% of specimens (mainly nymphs) of this tick species were active when the differences were in the range of 0-2.0%.

Hubalek *et al*. [27] found a significant effect of the amplitude in the temperature between soil and air on the activity of *I. ricinus* nymphs and adults as well as a lack of such a correlation in *Haemaphysalis concinna* nymphs and adults and in *D. reticulatus* adults in South Moravia (Czech Republic).

As in our previous study carried out during the tick seasonal activity period in eastern Poland, we did not find dependence between the level of humidity on the soil surface and the questing behaviour of *D. reticulatus* females and males during the winter activity. However, the delta of humidity between the two measurement points had a significant impact on stimulation of the locomotor activity in males, as its increase was correlated with the increased numbers of active males. The results of these observations indicate presence of physiological differences between the unfed specimens in both gender groups, which determine the capacity of maintenance of favourable water balance and the dynamics of active absorption of water vapour from the atmosphere.

The differences between the reactions of *D. reticulatus* females and males to the thermal and humidity stimulus may also be related to the different degree of body chitinisation and integumental permeability to water.

In questing ticks, elevated metabolism levels and ventilation through spiracles have been reported [28]. According to Gaede and Knülle [29], at high humidity, ticks can recompense any water deficit by active absorption using a hyperosmotic secretion from the salivary glands.

The absence of a correlation between the photoperiod and the activity of adult *D. reticulatus* ticks in our investigations is caused by the small differences in the day length during the collection period. Previous observations of seasonal rhythms of activity of this species demonstrated increased numbers of active females and males during a short day. In eastern Poland, diapause in adult *D. reticulatus* began when the day length was between 9h25’ and 8h53’ [19].

Autumn activity of adult *D. reticulatus* stages in the population from eastern Poland is 2 to 3 times higher than spring activity [19,30]. During the autumn activity, the largest numbers of questing ticks were observed between the second decade of September and the end of October or mid- November. The diapause of this tick species begins, depending on the climatic conditions, between mid-November and early December and lasts until March [19].

The winter occurrence of active *D. reticulatus* ticks in this study, which had never been reported during the previous long-term field studies in eastern Poland, may be attributed to weather changes, e.g. elevated temperature, a greater number of sunny days, and shorter persistence of the snow cover [24]. The mean temperature in December 2011 was above zero (1 to 2°C) and differed from the mean temperatures in December 2010 (-5 to -4°C) and 2012 (-4 to -3°C).

According to Nosek [1] and Bogdaszewska [23], the lower thermal threshold of activity of adult *D. reticulatus* ticks is 1°C. Hubalek *et al*. [27] reported questing of the tick species at air/soil temperatures of 0.7/-0.1°C. Based on investigations of *Dermacentor variabilis*, other authors have emphasized that, although it starts at the temperature rise, seasonal activity is not directly related to the daily temperature, but rather to increasing solar radiation [31]. As shown by our research, the response of *D. reticulatus* to a particular photoperiod is modified not only by temperature, but also by other conditions prevailing in the microhabitat, e.g. the snow cover. At lower temperatures, which limit the absorption of atmospheric water, ticks enter diapause earlier [19]. The ambient temperature plays a role in initiation and length of diapause by changing the dynamics of tick metabolism. Diapause length depends on the quantity of nutrient reserves accumulated during the consecutive tick developmental stages [32].

Similar to our study, high activity of nymphs and adult forms of another exophilic species (the European tick *I. ricinus*) was reported near Berlin (Germany). This was observed at minimum temperatures in January and February of 0.6 and 2.3°C, respectively, and mean temperature of ca. 7°C, i.e. in the typical period of its diapause in the temperate zone [33]. It is suggested that global warming and weather conditions can contribute to expansion of tick ranges and to changes in the dynamics of their activity and seasonal patterns of tick-borne diseases [30,34-43].

## Conclusions

The occurrence of active *D. reticulatus* ticks during the winter months in the temperate zone implies a potentially high risk of animal infestations and transmission of tick-borne diseases during weather changes during this period.

Among abiotic factors, soil and air temperature probably play the most important role in activation of *D. reticulatus* females, which have the greatest epidemiological importance due to their wide host range and feeding physiology.

The differences in the reaction of *D. reticulatus* females and males to temperature and humidity changes may be associated with the different structure of their integument and differences in physiological traits between specimens of the two genders determining maintenance of favourable water balance.

## Competing interests

The authors declare that they have no competing interests.

## Authors' contributions

AB designed the study, contributed to analysis and interpretation of obtained data, drafted the initial form of the manuscript, and reviewed comments by co-authors; KB contributed to interpretation of the data and to the statistical design and analysis plan and participated in writing of the manuscript. ZZ conducted statistical analyses and contributed to acquisition of data. All authors read and approved the final manuscript.
